# Defining RASopathy

**DOI:** 10.1242/dmm.049344

**Published:** 2022-02-01

**Authors:** Katherine A. Rauen

**Affiliations:** 1 Department of Pediatrics, Division of Genomic Medicine, University of California Davis, Sacramento, CA 95817, USA; 2 Department of Pediatrics, UC Davis MIND Institute, Sacramento, CA 95817, USA

## Abstract

The term RASopathy was originally created to describe a phenotypically similar group of medical genetic syndromes caused by germline pathogenic variants in components of the RAS/mitogen-activated protein kinase (RAS/MAPK) pathway. In defining a RASopathy syndrome, one needs to consider the complex nature of the RAS/MAPK pathway, the numerous genes and regulatory components involved, its crosstalk with other signaling pathways and the phenotypic spectrum among these syndromes. Three main guiding principles to the definition should be considered. First, a RASopathy is a clinical syndrome with overlapping phenotypic features caused by germline pathogenic variants associated with the RAS/MAPK pathway. Second, a RASopathy is caused by multiple pathogenetic mechanisms, all of which lead to a similar outcome of RAS/MAPK pathway activation/dysregulation. Finally, because a RASopathy has dysfunctional germline RAS/MAPK pathway activation/dysregulation, it may, therefore, be amenable to treatment with pathway modulators.

## Abstract (precis)

**Summary:** RASopathy is a term used to unify a phenotypically similar group of medical genetic syndromes, yet its definition is challenging and should consider the perspectives of clinicians, scientists and the patients.

## Introduction

**Fig. 1.**
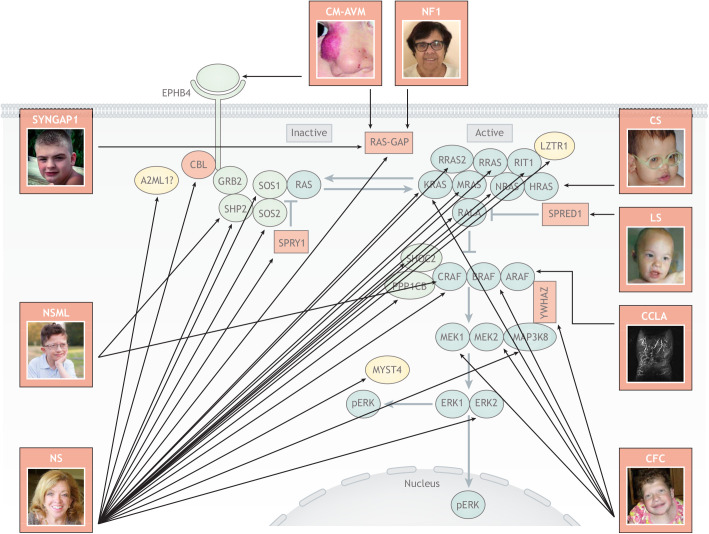
**The complexity of the RASopathies.** The RASopathies are a group of medical genetics syndromes that are caused by germline pathogenic variants in genes that encode components of the RAS/MAPK signal transduction pathway. The RASopathies are compiled of separate syndromes, and black arrows indicate the causative pathway component(s) for each. All pathogenic variants, whether they cause loss of function or protein activation, result in increased phosphorylation of ERK1 and/or ERK2 (pERK). Neurofibromatosis type 1 (NF1) is caused by numerous types of heterozygous mutations in the gene neurofibromin 1, resulting in haploinsufficiency of neurofibromin, a well-studied RAS-GAP. Shown is a 67-year-old female with a c.1541_1542delAG mutation. Image courtesy of the RASopathies Network. This image is not published under the terms of the CC-BY license of this article. For permission to reuse, please see the RASopathies Network. Capillary malformation arterio-venous malformation syndrome (CM-AVM) can be caused by mutations in the receptor tyrosine kinase gene *EPHB4* or the RAS-GAP gene *RASA1*. Shown is an image from Eerola and colleagues (Eerola et al., 2003) of an infant with a RASA1c.1579_1582delGTCT exon 11 deletion. This image is not published under the terms of the CC-BY license of this article. For permission to reuse, please see Eerola et al. (2003). Costello syndrome (CS) is one of the rarer RASopathies and is caused by heterozygous activating mutations in the canonical GTPase gene *HRAS*. Shown is a 4-year-old male with the common HRAS p.G12S pathogenic variant who developed a large abdominal rhabdomyosarcoma and passed away at age 7. Image courtesy of the RASopathies Network. This image is not published under the terms of the CC-BY license of this article. For permission to reuse, please see the RASopathies Network. Central conducting lymphatic anomalies (CCLA) can be caused by heterozygous pathogenic variants in the gene *ARAF*. Shown is an image of a T2-weighted noncontrast chest lymphangiogram of a 12-year-old male who harbors an activating ARAF p.S214P germline mutation, as reported by Li and colleagues (Li et al., 2019). This image is not published under the terms of the CC-BY license of this article. For permission to reuse, please see Li et al. (2019). Legius syndrome (LS) is caused by heterozygous loss-of-function mutations in SPRED1, which is an important negative regulator of the RAS-RAF interaction. Shown is a 3-year-old male with a SPRED1 p.R24X nonsense mutation, reported by Brems and colleagues (Brems et al., 2007). This image is not published under the terms of the CC-BY license of this article. For permission to reuse, please see Brems et al. (2007). Cardio-facio-cutaneous syndrome (CFC) is a rare RASopathy and caused by mutations in the RAS/MAPK pathway, which include KRAS, BRAF, MEK1, MEK2 and possibly YWHAZ, part of the 14-3-3 family of proteins. Shown is an 18-year-old female with a BRAF p.F468S missense mutation who passed away at 25 years old due to several complications during a surgical hospitalization. Image courtesy of the RASopathies Network. This image is not published under the terms of the CC-BY license of this article. For permission to reuse, please see the RASopathies Network. Noonan syndrome (NS) is the most common RASopathy and is caused by numerous genes encoding various components and regulators of the RAS/MAPK pathway, of which *PTPN11* is the most commonly mutated. Shown is a 54-year-old female who has a SHP2 p.D106A (protein product of *PTPN11*). Image courtesy of the RASopathies Network. This image is not published under the terms of the CC-BY license of this article. For permission to reuse, please see the RASopathies Network. Noonan syndrome with multiple lentigines (NSML) can be caused by two genes of the RAS/MAPK pathway, *PTPN11* or *RAF1*/*CRAF*. Shown is a 10-year-old boy with a heterozygous SHP2 p.T468M missense mutation. Image courtesy of the RASopathies Network. This image is not published under the terms of the CC-BY license of this article. For permission to reuse, please see the RASopathies Network. SYNGAP1 syndrome is rare RASopathy and caused by heterozygous missense or frameshift mutation in the RAS-GAP gene *SYNGAP1*. Shown here is a 13-year-old male with a heterozygous SYNGAP1 p.R1240X pathogenic variant. Image courtesy of the SYNGAP1 Foundation. This image is not published under the terms of the CC-BY license of this article. For permission to reuse, please see the SYNGAP1 Foundation.

The term RASopathy was created to describe, in a unifying fashion, a phenotypically similar group of medical genetic syndromes in which individuals have germline pathogenic variants in components of the RAS/mitogen-activated protein kinase (RAS/MAPK) pathway (Rauen, 2013; Tidyman and Rauen, 2009). Collectively, the RASopathies represent one of the largest groups of multiple congenital anomaly syndromes known. Noonan syndrome (NS) or an NS spectrum can be caused by a mutation in numerous genes, including *PTPN11*, *SOS1*, *RAF1*/*CRAF*, *KRAS*, *NRAS*, *SHOC2*, *CBL*, *RRAS*, *RIT1*, *RASA2*, *SOS2*, *MAP3K8*, *SPRY1*, *MYST4*/*KAT6B*, *LZTR1*, *A2ML1*, *PPP1CB*, *MRAS*, *RALA*, *RRAS2* and *ERK2*/*MAPK1.* Cardio-facio-cutaneous syndrome (CFC) is caused by activating mutations in multiple genes, including *BRAF*, *MAP2K1*/*MEK1*, *MAP2K2*/*MEK2*, *KRAS* and possibly *YWHAZ.* Other RASopathies, including neurofibromatosis type 1 (NF1), Noonan syndrome with multiple lentigines (NSML), Costello syndrome (CS), Legius syndrome, SYNGAP1 and central conducting lymphatic anomalies (CCLA), are caused by alterations in one or two genes in the RAS pathway (Fig. 1) (Tidyman and Rauen, 2016). Thus far, all causative pathogenic variants are part of the complex and well-studied RAS/MAPK signal transduction pathway, which is essential for development and is implicated in oncogenesis. What makes the RASopathies unique is the pathway-based, mechanistic approach to defining medical genetic syndromes as opposed to the isolated one gene–one syndrome approach.

**Figure:**
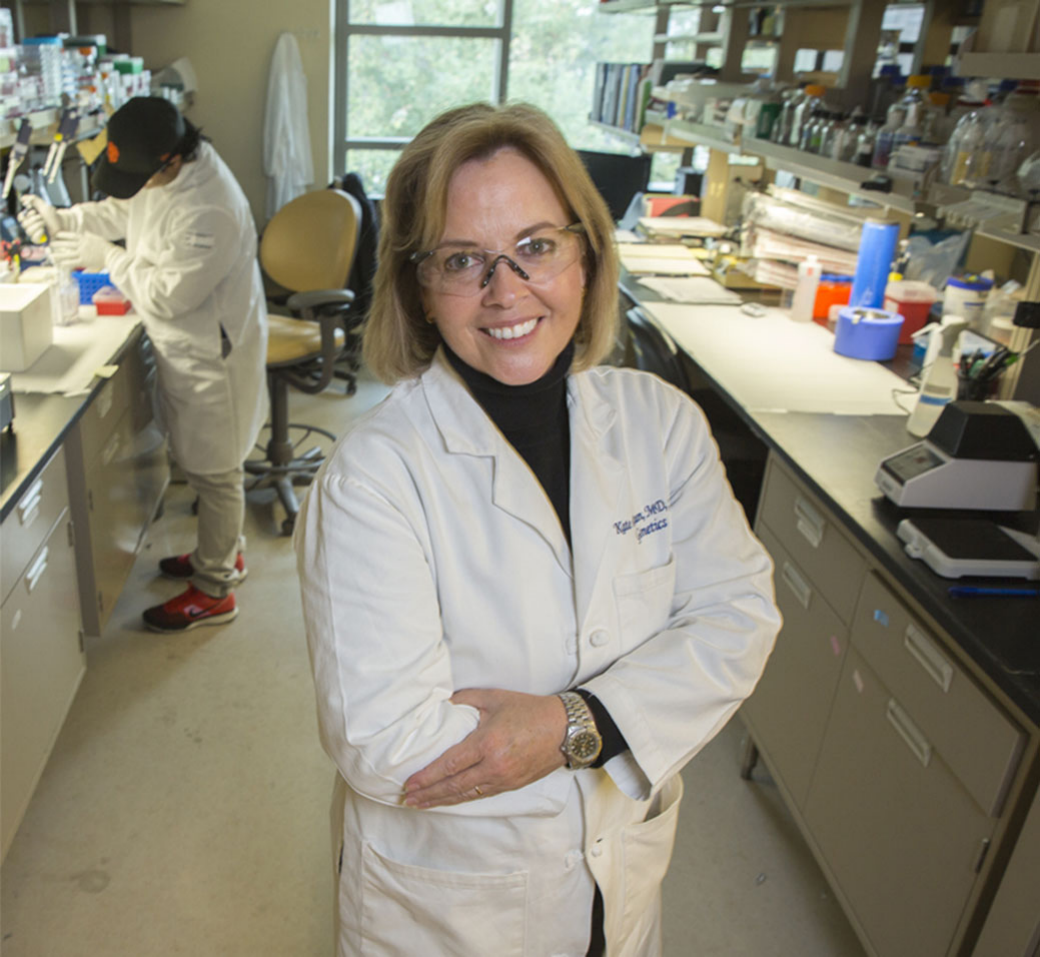
Katherine A. Rauen

Functional studies used to identify the pathogenicity of a RASopathy gene variant have relied on biochemical analysis or animal modeling to demonstrate ERK activation/dysregulation, which is the final MAPK effector of the RAS/MAPK pathway, with phosphorylated ERK1/2 having a plethora of nuclear and cytosolic targets. It has been difficult to concisely define RASopathies because of the complex nature of RAS/MAPK pathway signaling, the numerous regulators of the pathway, the ever-increasing number of genes that influence its activity and the sheer number of RASopathies that exist. As such, a clear consensus on the definition of a RASopathy is lacking, and perhaps this difficulty is enhanced because the term has different meanings depending on one's perspective.

“All causative pathogenic variants are part of the complex and well-studied RAS/MAPK signal transduction pathway […] What makes the RASopathies unique is the pathway-based, mechanistic approach to defining medical genetic syndromes as opposed to the isolated one gene–one syndrome approach.”

## Genomic medicine perspective

From a genomic medicine perspective, the RASopathies are a group of multiple congenital anomaly syndromes that have a recognizable and overlapping clinical phenotype. It is these recognizable phenotypic features, along with associated anomalies, that clinical geneticists have traditionally considered when establishing a clinical diagnosis of a RASopathy syndrome. It is important to note that RASopathies are dynamic syndromes, in that phenotypic findings in the newborn are different from those findings seen in childhood, adolescence and adulthood, therefore making a clinical diagnosis challenging. In addition, RASopathy-associated gene variants often occur *de novo*, as well as are inherited, and mosaicism may occur (Jafry and Sidbury, 2020).

Having now examined thousands of RASopathy patients, phenotypically, RASopathy syndromes have craniofacial features that are similar to features seen in NS, the most common of the RASopathies (Fig. 1). Craniofacial features among NS, CS, CFC, Legius syndrome and even NF1 can be so similar that it is difficult to clinically distinguish these syndromes in the newborn period (for a comprehensive review including phenotypic features, see Tidyman and Rauen, 2016). Cardiovascular anomalies are also a common feature among RASopathies. The vast majority of individuals with CS, CFC, NS and NSML have cardiac defects. Although rarer, cardiac issues may also be seen in NF1 (Friedman et al., 2002) and Legius syndrome (Brems et al., 2007). Skin abnormalities are a common unifying feature of RASopathies, implicating dysregulation during neural crest development (Brannan et al., 1994). NF1 has the most recognizable skin phenotype, with its clinical diagnosis relying on cutaneous criteria (Legius et al., 2021). However, NSML, CFC and CS also have unique cutaneous findings that may aid in the clinical diagnosis (Bessis et al., 2019; Siegel et al., 2012, 2011). Lymphatic issues are understudied in RASopathies but can cause significant morbidity and early mortality. Lymphatic anomalies were solidified as an important phenotypic feature when *ARAF* pathogenic variants, along with other RASopathy-associated genes, were recently identified in CCLA, a newly identified RASopathy (Li et al., 2019). The lymphatic abnormalities were rescued by the use of an MEK inhibitor, which supports the underlying mechanistic pathway approach to RASopathies. Vascular anomalies are also found in RASopathies. NF1 was the first identified to have vascular malformations including renal artery stenosis (Halpern and Currarino, 1965), but, upon close examination, other RASopathies can have vascular malformations as well (Eerola et al., 2003). The most striking is capillary malformation arterio-venous malformation syndrome (CM-AVM), in which the phenotype is defined by vascular malformations and not by dysmorphic craniofacial features or developmental delay, highlighting the importance of tissue-specific, temporal-spatial expression of RASopathy-causing genes during development (Eerola et al., 2003). However, although not systematically studied, NS, CFC, CS and Legius syndromes are also known to have vascular malformations. Neurocognitive and behavioral issues are very common in RASopathies. Syndromes have markedly varying degrees of neurocognitive delay ranging from normal IQ to severe developmental delay (Tidyman and Rauen, 2009). Behavioral issues such as hyperactivity, attention deficient, defiant behavior, depression, anxiety, sleep disturbances as well as autism significantly impact the RASopathies (Adviento et al., 2014; Alfieri et al., 2014; Rauen et al., 2018). SYNGAP1 is a RASopathy that does not have the typical craniofacial features, cardiac issues or skin abnormalities, but has many of the overlapping neurocognitive, behavioral and autistic features found in RASopathies due to the unique neuronal restricted expression of SYNGAP1 (Hamdan et al., 2009). It is important to note, from a clinical perspective, that a RASopathy should not be solely defined based on the presence of a craniofacial phenotype, which has traditionally been the case with clinical geneticists. Craniofacial features may be only one aspect of a RASopathy, as the full phenotype can encompass other issues including cardiac, lymphatic, vascular, cutaneous, neurocognition, neurodevelopmental, behavioral, orthopedic, ocular, auditory, neuromuscular, gastrointestinal and neoplasia.

A clinical diagnosis is often difficult even for a RASopathy expert because of the overlapping RASopathy phenotype. Syndromic boundaries have become blurred as the phenotypic spectrum has expanded, making molecular diagnosis essential. At the present time, RASopathies are categorized as separate syndromes, but, as we learn more about each syndrome, clinically defining a RASopathy may be more accurately approached based on the molecular diagnosis, or gene name, such as a *BRAF*-based RASopathy or an *HRAS*-based RASopathy. Moreover, with the increased use of whole-exome/genome sequencing in genomic medicine, additional genes will be identified that cause RAS/MAPK pathway dysregulation and are associated with a RASopathy phenotype; however, the individual may not necessarily present with classical phenotypic features of a well-defined RASopathy. The focus of a clinical geneticist is making a correct diagnosis, which is essential to managing the health care of the patient. However, the overall phenotypic approach, although important, is becoming potentially antiquated, and it may be more informative to establish the molecular etiology of the suspected RASopathy rather than try to precisely force a clinical diagnosis into an existing phenotypically named syndrome.

“The overall phenotypic approach, although important, is becoming potentially antiquated, and it may be more informative to establish the molecular etiology of the suspected RASopathy rather than try to precisely force a clinical diagnosis into an existing phenotypically named syndrome.”

## RAS biologist perspective

Having had the privilege of working closely with some of the pre-eminent experts in RAS biology and having introduced them to the RASopathies, it is clear that their perspective of the RASopathies is grounded in their depth of knowledge regarding the complexity of the RAS/MAPK pathway. Our knowledge of its detail is continually increasing, including the role and interaction of pathway proteins in regulatory networks, crosstalk with other intracellular signaling pathways, feedback loops, scaffolding proteins and downstream effector targets. The focus of the RAS biologist is on the mechanisms by which gene variants associated with RASopathy syndromes affect RAS/MAPK signaling (Fig. 1). RAS proteins are small guanosine nucleotide-bound GTPases that act as a central signaling hub for multiple intracellular downstream effector pathways (Simanshu et al., 2017). RAS genes exist as a large multigene family, with the canonical *HRAS*, *NRAS* and *KRAS* being the best studied. All three of these RAS proteins have been identified as mutated in RASopathies, along with non-canonical RAS proteins, RIT1, MRAS, RRAS, RRAS2 and RALA (Tidyman and Rauen, 2016). RAS then signals to numerous downstream effector pathways via serine/threonine kinase RAF (ARAF, BRAF and CRAF/RAF1), which, when dysregulated in the germline, causes RASopathies. The identification of new gene variants has provided RAS researchers with information on heretofore unknown genes linked to the RAS/MAPK pathway. For example, mutations in *LZTR1*, which causes NS, provided the clue that led to the discovery that LZTR1 interacts with RIT1 and perhaps other RAS family members, thereby dysregulating the RAS/MAPK pathway (Castel et al., 2019).

From a pathway perspective, the RASopathies have highlighted that functional dysregulation of the RAS/MAPK pathway can occur by multiple mechanisms. RASopathy germline mutations have been identified in receptor tyrosine kinases, GTPases, RAS GTPase-activating proteins (RAS-GAPs), RAS guanine nucleotide exchange factors (RAS-GEFs), kinases, scaffolding/adaptor proteins, ubiquitin ligases, phosphatases and pathway inhibitors – with all identified mutations culminating in the activation of ERK1/2 (Tidyman and Rauen, 2016). Although RAS signals to multiple intracellular effector cascades (Klomp et al., 2021), the central driving pathogenetic alteration underlying all of the RASopathies is RAS/MAPK pathway activation/dysregulation. A large portion of observed RASopathy mutations affects components upstream of RAS, resulting in aberrant RAS activation. However, each syndrome results from mutations in different genes associated with the RAS/MAPK pathway. In addition, distinct mutations within each of these genes may affect RAS signaling differentially, thus resulting in phenotype variability. Even though the central molecular etiology is RAS/MAPK pathway activation/dysregulation, the complexity of temporal-spatial signaling to other pathways is also certain to play a significant role in the unique phenotypic features of RASopathies.

## Oncologist perspective

From an oncologist's perspective, the RASopathies are cancer syndromes due to the presence of potentially oncogenic mutations in the germline (Kratz et al., 2011). RAS/MAPK pathway dysfunction is implicated in cancer, with somatic RAS drivers being responsible for ∼20% of malignancies (Bos, 1989) and hyperactivated ERK being found in a third of human cancers due to various other mutations associated with the pathway (Hoshino et al., 1999). Germline pathogenic variants in both oncogenes and tumor suppressor genes occur in multiple RASopathies, with many being identical to those found somatically mutated in cancer. However, important functional differences exist. Many RASopathy activating mutations are novel, not found in cancer, and produce lower-level RAS/MAPK pathway activation compared to those associated with cancer. For example, the strongly activating BRAF p.V600E driver commonly present in melanoma, thyroid, ovarian and colorectal cancer has never been identified in a CFC individual (Champion et al., 2011; Rodriguez-Viciana et al., 2006), and KRAS mutations identified in a RASopathy individual are novel hypermorphs and not in the classic mutational hotspots associated with cancer (Schubbert et al., 2007). Thus, driver mutations in RAS and downstream pathway kinases that are present in rarer RASopathies, such as CS and CFC, may reflect intolerance for such mutations during development due to robust pathway activation.

The RASopathies provide a unique perspective in the study of cancer genes because individuals harbor a single germline mutation, unlike a somatic cancer, which typically acquires many oncogenic mutations. Understanding the function of RASopathy-associated genes in the RAS/MAPK pathway, and their role in development, provides clues as to its function in cancer and vice versa. NF1 is the best-studied cancer syndrome caused by germline haploinsufficiency of the tumor suppressor gene neurofibromin 1. NF1 individuals have more than twice the risk of cancer of the general population (Evans et al., 2017). Malignant peripheral nerve sheath tumors are rare in the general population but are one of the most common causes of early death in NF1 (Evans et al., 2011, 2017). Other cancers seen in NF1 include juvenile myelomonocytic leukemia (JMML), neuroblastoma, embryonal rhabdomyosarcoma, glioma, pheochromocytoma, gastrointestinal stromal tumors, somatostatinomas, breast cancer, acute lymphoblastic leukemia (ALL) and melanoma (Evans et al., 2017). NS individuals are also at higher risk for cancer, and many of the cancers seen in NF1 are also seen in NS, such as JMML, ALL, neuroblastoma, embryonal rhabdomyosarcoma, glioma and acute myeloid leukemia (Kratz et al., 2011). Additionally, ∼17% of CS individuals will develop cancer in their lifetime, with embryonal rhabdomyosarcoma being the most common, followed by transitional cell carcinoma and neuroblastoma (Gripp, 2005). Although more information is needed, an increased cancer risk may also be associated with CFC, NSML, CM-AVM, SYNGAP1 and Legius syndrome. Importantly, discovery of RASopathy-associated genes has led to the identification of new oncogenes. For example, MEK mutations had never been identified in cancer until we identified germline MEK1/2 mutations in CFC (Rodriguez-Viciana et al., 2006). Since then, activating MEK mutations have been identified in numerous cancers.

Oncology research and treatment has focused extensively on the function of the RAS/MAPK pathway, and, as such, the pathway presents an attractive target for utilizing small-molecule therapeutics to specifically inhibit the pathway. Small-molecule inhibitors originally developed to treat cancer, such as farnesyl transferase inhibitors, MEK inhibitors, ERK inhibitors and several more, provide opportunities to modulate pathway activity and therapeutically treat developmental disorders caused by germline RAS/MAPK hyperactivation (Dombi et al., 2016). As also shown in this Special Issue of DMM, we recently demonstrated normalization of a skeletal myopathy using a MEK inhibitor in an adult CS *Hras* mouse model (Tidyman et al., 2021). Because many of the developmental phenotypic signs and symptoms of RASopathies are not static, the possible use of systemic therapies after birth to reduce pathway activity holds the potential to ameliorate germline syndromic disease progression.

## Patient/parent perspective

In defining a RASopathy, one must consider the patient and family perspective. Having been closely involved with RASopathy patient advocacy groups, even before the molecular genetic causes had been identified, has provided a great deal of insight into the patient/parent perspective. Most families have been through a long, frustrating diagnostic odyssey, with many individuals initially being misdiagnosed. RASopathies may arise *de novo*; however, germline transmission may also occur, especially in NF1 and NS, two of the most common RASopathies. The diagnosis of a RASopathy is a life-altering event for many patients and their families. These are chronic disorders that can profoundly interfere with activities of daily living. Because RASopathies typically affect multiple organ systems, delay development, and alter behavior and neurocognition, it is common for patients to be supported by a health care team for medical management. Therefore, it is essential that patients are evaluated by specialists and have continued follow-up with a multidisciplinary team throughout their lifespan. The lifespan of individuals with RASopathies is unknown as this question has not been systematically evaluated; however, it is known that those with NF1 do have a shorter life span due to cancer morbidity. Recognizing the complex medical needs of a patient/family with a RASopathy, we launched the first pathway-based clinic, which is now emulated globally. From the patient and family perspective, the definition of a RASopathy includes the knowledge that even with the diagnosis of a specific syndrome that itself may be rare, the individual is now part of a large family of clinical syndromes with common underlying pathogenetic mechanisms. This knowledge has made patients and their families far less isolated and has encouraged a network of support with the sharing of medical management information and research gained from the various RASopathy syndromes (Gripp et al., 2020). Additionally, the pathway approach has sparked an interconnection of advocacy support groups such as the RASopathies Network, Costello Syndrome Family Network, NF Inc., CFC International, SYNGAP1 Foundation and others that provide much needed educational support and access to medical expertise for families and individuals.

“The diagnosis of a RASopathy is a life-altering event for many patients and their families. These are chronic disorders that can profoundly interfere with activities of daily living.”

## RASopathy definition

In summary, defining a RASopathy is not simple. One needs to take into consideration the complex nature of the RAS/MAPK pathway due to the numerous genes and regulatory components involved and its crosstalk with other signal transduction pathways, the expanse of knowledge yet to be uncovered and the phenotypic spectrum among the varying syndromes. For a syndrome to be considered a RASopathy, there are three main principles to the definition that should be considered. First, a RASopathy is a clinical syndrome with overlapping features caused by germline pathogenic variants. Phenotypic findings and congenital anomalies are wide ranging because the RAS/MAPK pathway is critical to development; however, an overarching pattern of phenotypic features does occur. Cardiac issues, vascular or lymphatic anomalies, delay in development with possible neurocognitive issues, failure to thrive in childhood, cutaneous findings and neoplasia/cancer are overlapping features that lead one to clinically suspect a RASopathy. Moreover, Noonan-like craniofacial dysmorphology alone does not define a RASopathy, nor does the lack of this phenotype preclude it, but rather it is a component of the phenotype that may lead one to suspect a RASopathy. Second, RASopathies may be caused by multiple pathogenetic mechanisms, all of which lead to a similar outcome – RAS/MAPK pathway activation/dysregulation. Therefore, a RASopathy is caused by a molecular pathogenic variant(s) within a gene(s) that encodes a component or regulator of the RAS/MAPK pathway, which causes the activation of ERK1 and/or ERK2. The pathogenic variant may be *de novo* or may be inherited. Finally, a RASopathy is a potential cancer syndrome that may predispose individuals to neoplasia, benign or malignant, within their lifetime, and, as such, the same small-molecular inhibitor that may be used to treat a somatic cancer caused by RAS/MAPK pathway activation, such as a MEK inhibitor, may be considered, with modification, to potentially treat germline phenotypic features of a RASopathy, such as cardiomyopathy, neurocognitive issues, lymphatic disorder, etc. It is understandable that nuisances to the definition exist, especially when one is defining a term that continues to evolve. Nonetheless, as our pathway knowledge increases and our understanding of the RAS/MAPK phenotypic spectrum becomes more refined, we should be mindful that RASopathies are a condition with great human impact and there is a face behind each RASopathy.
